# Role of the long non-coding RNAs in regulation of Gemcitabine response in tumor cells

**DOI:** 10.1186/s12935-023-03004-7

**Published:** 2023-08-14

**Authors:** Amirhosein Maharati, Yalda Samsami, Hanieh Latifi, Faezeh Tolue Ghasaban, Meysam Moghbeli

**Affiliations:** 1 Student Research Committee, Faculty of Medicine, Mashhad University of Medical Sciences, Mashhad, Iran; 2 Department of Medical Genetics and Molecular Medicine, School of Medicine, Mashhad University of Medical Sciences, Mashhad, Iran

**Keywords:** Long non-coding RNAs, Gemcitabine, Chemo resistance, Prognosis, Non-invasive marker, Cancer

## Abstract

Chemotherapy is widely used as one of the first line therapeutic methods in cancer patients. However, chemotherapeutic resistance is one of the most common problems in cancer patients, which leads to the therapeutic failure and tumor relapse. Considering the side effects of chemotherapy drugs in normal tissues, it is required to investigate the molecular mechanisms involved in drug resistance to improve the therapeutic strategies in cancer patients. Long non-coding RNAs (lncRNAs) have pivotal roles in regulation of cellular processes associated with drug resistance. LncRNAs deregulations have been frequently reported in a wide range of chemo-resistant tumors. Gemcitabine (GEM) as a nucleoside analog has a wide therapeutic application in different cancers. However, GEM resistance is considered as a therapeutic challenge. Considering the role of lncRNAs in the occurrence of GEM resistance, in the present review we discussed the molecular mechanisms of lncRNAs in regulation of GEM response among cancer patients. It has been reported that lncRNAs have mainly an oncogenic role as the inducers of GEM resistance through direct or indirect regulation of transcription factors, autophagy, polycomb complex, and signaling pathways such as PI3K/AKT, MAPK, WNT, JAK/STAT, and TGF-β. This review paves the way to present the lncRNAs as non-invasive markers to predict GEM response in cancer patients. Therefore, lncRNAs can be introduced as the efficient markers to reduce the possible chemotherapeutic side effects in GEM resistant cancer patients and define a suitable therapeutic strategy among these patients.

## Background

Surgery, radiotherapy, and chemotherapy are the most common therapeutic methods in cancer patients that can be used alone or in combination. Among these strategies, chemotherapy has been considered as the first-line therapeutic method that is widely applied in many cancers. Chemotherapy approaches are based on the chemical anti-cancer substances that affect either tumor or normal cells. Consequently, this non-specific function causes a wide range of adverse effects in cancer patients [1]. The main challenge of chemotherapy is drug resistance that is responsible for approximately 90% of treatment failures and tumor relapses [2]. Tumor relapse can be observed in more than half of the Non–small cell lung carcinoma (NSCLC) patients following the chemotherapeutic treatment [3]. There was also 50–70% of tumor recurrence after chemotherapy in ovarian cancer patients [4]. Therefore, precise identification of molecular mechanisms involved in drug resistance is necessary to improve the chemotherapeutic efficacy. Genetic mutations, epigenetics alterations, drug efflux, DNA repair, epithelial–mesenchymal transition (EMT), and tumor microenvironment have been associated with drug responses in tumor cells [2, 5, 6]. It is noteworthy that more information about the molecular mechanisms of drug resistance can be useful to present the appropriate prognostic markers to minimize the tumor relapse and side effects. However, due to the heterogeneity among tumor cells and tissues, determining a better strategy will be challenging [7]. Gemcitabine (GEM) is a chemotherapy drug that has been approved for the treatment of late-stage pancreatic cancer (PC). However, it is currently used as an adjuvant therapy in various solid tumors. GEM acts as a deoxycytidine analog that prevents DNA synthesis, thereby promoting apoptosis in malignant tumor cells [8, 9]. Although, GEM is a common drug in cancer patients that significantly improves the overall survival, GEM resistance is still considered as a big challenge among a noticeable rate of cancer patients [10]. Different transcription factors, molecular mechanisms, signaling pathways, and metabolic enzymes are involved in GEM response [8]. Long non-coding RNAs (lncRNAs) are a group of the non-coding RNAs that modulate the transcription or translation of target genes [11]. They have pivotal functions in various cellular processes such as cell proliferation, differentiation, and migration through interaction with proteins, DNA, or RNA [12]. LncRNAs function as competing endogenous RNAs (ceRNAs) to affect miRNAs functions and their target genes. They can also directly bind with the DNA and transcription factors to repress and promote gene expressions, respectively [13]. Deregulation of lncRNAs is correlated with tumor initiation and progression as either oncogenes or tumor suppressors [14–18]. As the lncRNAs are extensively participated in various physiological processes and tumorigenesis, aberrant expression of lncRNAs can be associated with chemotherapy resistance [19]. LncRNAs induce the expression of genes that are associated with drug resistance via enhancing proliferation while reducing apoptosis in various cancers [20]. Role of lncRNAs in GEM resistance has been frequently reported in different cancers [21–23]. According to the presence of lncRNAs in blood samples, they can also be suggested as reliable non-invasive prognostic and diagnostic indicators in cancer patients. Therefore, in the present review we discussed the role of lncRNAs in regulation of GEM response to present them as the probable efficient non-invasive prognostic markers in cancer patients (Table 1).

**Table 1:** LncRNAs that are involved in Gemcitabine (GEM) response in tumor cells

Study	Year	Tumor Type	LncRNA	Target	Samples	Results	Clinical Application
Gao (8)	2021	Non-small cell lung	MEG3	PTEN	A549 and H520 cell lines Xenograft models	Decreased GEM resistance	Diagnosis
Sun (29)	2019	Osteosarcoma	PVT1	miR-152/c-MET	MG63 and 293 T cell lines Xenograft model	Increased GEM resistance	Diagnosis
Hong (34)	2020	Colorectal	AGAP2-AS1	miR-497/ FGFR1	116T 116 N DLD-1, SW480, HT29, CaCO2, RKO, HCT8 and 293T cell lines Xenograft model	Increased GEM resistance	Diagnosis and prognosis
Xu (37)	2021	Pancreatic	HIF1A-AS1	HIF1a	69T(24 GEM-sensitive) BxPC3 and PANC1 cell lines Xenograft model	Increased GEM resistance	Diagnosis and prognosis
Pan (39)	2016	Bladder carcinoma	UCA1	miR-196a-5p/CREB	35T 18 N 5637 and UMUC-2 cell lines Xenograft model	Increased GEM resistance	Diagnosis
Xiong (46)	2019	Pancreatic	GSTM3TV2	Let-7/LAT2	180T 180 N AsPC-1/GR and MIAPaCa-2 cell lines Xenograft model	Increased GEM resistance	Diagnosis and Prognosis
Xu (49)	2021	Pancreatic	DLEU2L	BRCA2	PANC-1 cell line Xenograft model	Decreased GEM resistance	Diagnosis
Liu (53)	2019	Pancreatic	HCP5	miR-214-3p/HDGF	28T 28 N PANC-1 and SW 1990 cell lines Xenograft model	Increased GEM resistance	Diagnosis and prognosis
Zhou (59)	2020	Pancreatic	PVT1	miR-619/Pygo2	PANC-1 and ASPC1 human cell lines Xenograft model	Increased GEM resistance	Diagnosis and prognosis
Lu (60)	2021	Cholangiocarcinoma	LINC00665	miR-424-5p/BCL9L	100T 100 N HuCCT1, HuH28, SNU-1196, SNU-1079, SNU-308, SNU245, SNU-478, SNU-869 and HEK293T cell lines Xenograft model	Increased GEM resistance	Diagnosis and prognosis
Xie (61)	2018	Bladder	CDKN2B-AS	let-7/ CTNNB1	81T 34 N SV-HUC-1, J82 and T24 cell lines	Increased GEM resistance	Diagnosis and prognosis
Yu (66)	2022	Pancreatic	SNHG16	SMAD4	SW1990, PANC-1, ASPC-1, BxPC3 and HPDE cell lines	Increased GEM resistance	Diagnosis
Zhuang (73)	2017	Bladder	LET	NF90 and miR-145	60T 48 N T24, 5637, J82, SW780, BIU87, ScaBER and UMUC3 cell lines Xenograft model	Decreased GEM resistance	Diagnosis and prognosis
Liu (75)	2018	Pancreatic	GAS5	miR-221/ SOCS3	60T 60 N HPDE6-C7 s PANC-1, AsPC-1, Capan-2, SW1990 and BXPC-3 cell lines Xenograft model	Decreased GEM resistance	Diagnosis and prognosis
Chi (76)	2021	Pancreatic	UCA1	SOCS3	35T 35 N Human PSC, PANC-1 and HEK-293T cell lines Xenograft model	Increased GEM resistance	Diagnosis
Shen (85)	2020	Cholangiocarcinoma	LINC01714	FOXO3	70T 70 N HuCCT1 and CCLP1 cell lines Xenograft model	Decreased GEM resistance	Diagnosis and prognosis
Shi (92)	2019	Pancreatic	LINC00346	miR-188-3p/ BRD4	24T PANC-1, MIA PaCa-2, Capan-1 and BxPC-3 cell lines Xenograft model	Increased GEM resistance	Diagnosis
Li (94)	2015	Pancreatic	HOTTIP	HOXA13	90T PANC-1, MIA PaCa-2, Capan-2, SW1990, and BxPC-3 cell lines Xenograft model	Increased GEM resistance	Diagnosis
Wang (96)	2021	Pancreatic	ANRIL	miR-181a/ HMGB1	PANC-1, BxPC-3 and HPDE cell lines	Increased GEM resistance	Diagnosis
Xue (124)	2020	Gallbladder	SSTR5-AS1	NONO	110T 110 N GBC-SD, SGC-996 and NOZ cell lines Xenograft model	Increased GEM resistance	Diagnosis and prognosis
Zhang (130)	2019	Pancreatic	SNHG14	miR-101	SW1990 cell line	Increased GEM resistance	Diagnosis
Chen (137)	2016	Breast	ROR	miR-34a	MDA-MB-231 and MCF10A cell lines	Increased GEM resistance	Diagnosis
An (138)	2020	Pancreatic	HOST2	-	BxPC-3, CFPAC-1, SU.86.86, PANC-1, Hs766T and AsPC-1 cell lines	Increased GEM resistance	Diagnosis
Sun (141)	2019	Pancreatic	MSC-AS1	miR-29b-3p/ CDK14	45T 45 N PANC-1 and BxPC-3 cell lines	Increased GEM resistance	Diagnosis and prognosis
Li (142)	2019	Bladder	GHET1	ABCC1	74T(41 GEM-sensitive) J82, T24, SV-HUC-1 J82/Gem and T24/Gem cell lines	Increased GEM resistance	Diagnosis and prognosis
An (143)	2018	Bladder	FOXD2-AS1	miR-143/ ABCC3	T24 and 5637 cell lines Xenograft model	Increased GEM resistance	Diagnosis
Yang (150)	2020	Pancreatic	SLC7A11-AS1	NRF2	27T 27 N BxPC-3, PANC-1 and AsPC-1 cell lines Xenograft model	Increased GEM resistance	Diagnosis and prognosis
Ye (151)	2022	Pancreatic	DBH-AS1	miR-3163/ USP44	172T 16 N HPDE and PC cell lines Xenograft model	Decreased GEM resistance	Diagnosis and prognosis
Hua (154)	2019	Pancreatic	SBF2-AS1	miR-142-3p/ TWF1	82T 82 N AsPC-1, HPAC, BxPC-3 and PANC-1 cell lines	Increased GEM resistance	Diagnosis and prognosis
Xu (157)	2019	Gastric	MVIH	E-cadherin and Vimentin	Human BGC-823 cell lines	Decreased GEM resistance	Diagnosis

* Tumor (T) tissues and Normal (N) margins

## PI3K/AKT and MAPK signaling pathways

**Figure 1:**
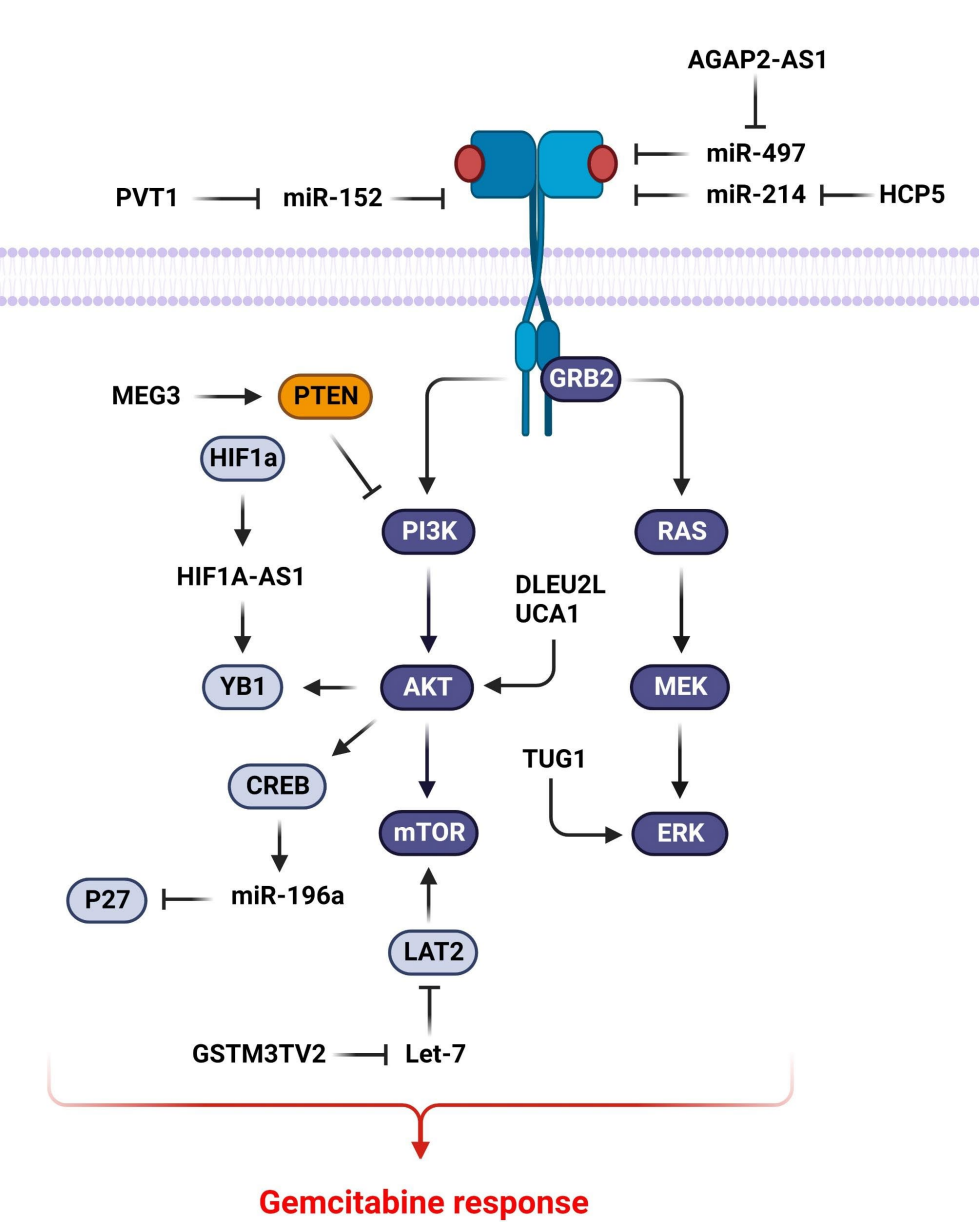
LncRNAs have a key role in GEM response of tumor cells by regulation of PI3K/AKT and MAPK/ERK signaling pathway. (Created with BioRender.com)

PI3K/AKT is an important signaling pathway that can be activated by growth factors following the binding with receptor tyrosine kinases (RTKs). AKT is the most important downstream effector of PI3K that has pivotal roles in cellular metabolism, growth, and proliferation. Therefore, deregulation of PI3K/AKT pathway can be associated with drug resistance and tumor relapse [24]. LncRNAs have a key role in GEM response of tumor cells by regulation of PI3K/AKT signaling pathway (Fig. 1). Surgery, chemotherapy, and radiotherapy are the main therapeutic plans in osteosarcoma patients. A standard chemotherapy regimen including cisplatin, GEM, and doxorubicin can improve the 5-years survival rate of osteosarcoma patients [25]. However, drug resistance is still a big challenge for the effective treatment of osteosarcoma patients [26]. C-MET is a receptor tyrosine kinase that increases tumor progression in a wide range of tumors [27]. A study has reported that c-Met inhibition increased the sensitivity of osteosarcoma cells to cisplatin by PI3K/Akt suppression [28]. PVT1 activated the PI3K/AKT pathway via c-MET to increase the chemotherapy resistance of osteosarcoma cells. PVT1 had a pivotal role in the GEM resistance of osteosarcoma cells through miR-152 targeting to regulate the c-MET/PI3K pathway [29]. FGFR1 belongs to the RTK protein family that promotes MAPK and PI3K/Akt pathways [30]. FGFR1 activation has been associated with EMT process in several human cancers [31–33]. AGAP2-AS1 up regulation was correlated with advanced tumor stage and poor survival in CRC patients. AGAP2-AS1 sponged miR-497 to promote the growth and metastasis of CRC cells and GEM resistance via FGFR1 targeting [34]. YB1 as a highly conserved transcription factor is involved in regulation of a wide variety of biological processes [35]. The YB-1 phosphorylation by AKT triggers the mRNA translation [36]. PI3K/AKT over-activation is involved in GEM resistance. HIF1A-AS1 modulated the HIF1a expression in a glycolysis-dependent manner to promote GEM resistance in pancreatic tumor cells. HIF1A-AS1 improved the interaction between p-AKT and p-YB1 to induce YB1 phosphorylation. YB1 phosphorylation by AKT plays a pivotal role in the progression of GEM resistance in PC via HIF1a regulation by HIF1A-AS1. There was also a significant association between HIF1A-AS1/HIF1a up regulation and the poor prognosis in GEM-received PC patients [37].

Drug resistance restricts the long-term therapeutic impacts of the cisplatin and GEM in bladder cancer treatment [38]. UCA1 induced cisplatin/GEM resistance in bladder tumor cells by CREB mediated miR-196a-5p regulation. UCA1 triggered the AKT pathway that resulted in CREB phosphorylation. UCA1 also increased cisplatin/GEM resistance via miR-196a-5p up regulation by CREB and p27Kip1 regulations in bladder cancer [39]. Although, GEM is an effective and widely prescribed therapy for NSCLC patients, GEM resistance has restricted its clinical application. Curcumin has been indicated to play a tumor suppressive role by regulation of the signaling pathways in various cancers [40, 41]. Curcumin promotes apoptosis and autophagy in lung tumor cells via STAT3 and PI3K/AKT pathways [42, 43]. PTEN as a negative regulator of PI3K/AKT pathway has a key role in various tumor associated phenotypes such as relapse and drug response. It has been shown that high concentrations of curcumin enhanced the apoptosis of GEM-resistant NSCLC cells. Curcumin up regulated the MEG3 that promoted the PTEN pathway in GEM-resistant NSCLC cells. Curcumin also suppressed the GEM-resistant NSCLC proliferation in the xenograft model [8]. The mTOR is the main downstream target of PI3K/Akt that induces cell growth and protein synthesis by activation of S6K and 4EBP1 [44]. LAT2 is a membrane transporter involved in mTOR activation to induce chemo resistance in PC cells [45]. GSTM3TV2 up regulated the LAT2 by competitively sponging let-7, resulting in increased GEM resistance in pancreatic tumor cells. GSTM3TV2 up regulation was associated with poor prognosis in PC patients [46]. BRCA2 prevents mutagenesis as a tumor-suppressor via regulating DNA double-strand break repair [47, 48]. GEM is a nucleoside analog that suppresses DNA replication in tumor cells. It has been found that the DLEU2L down regulated the Warburg effect modulators including GLUT1, LDHB, HK2, and PKM2 in PC cells. DLEU2L decreased ATP production and glucose uptake. It also inhibited the phosphorylation of AKT/mTOR, as well as S6K as downstream effectors. Moreover, DLEU2L inhibited GEM resistance in PC cells via miR-210-3p/BRCA2 axis [49].

**Figure 2:**
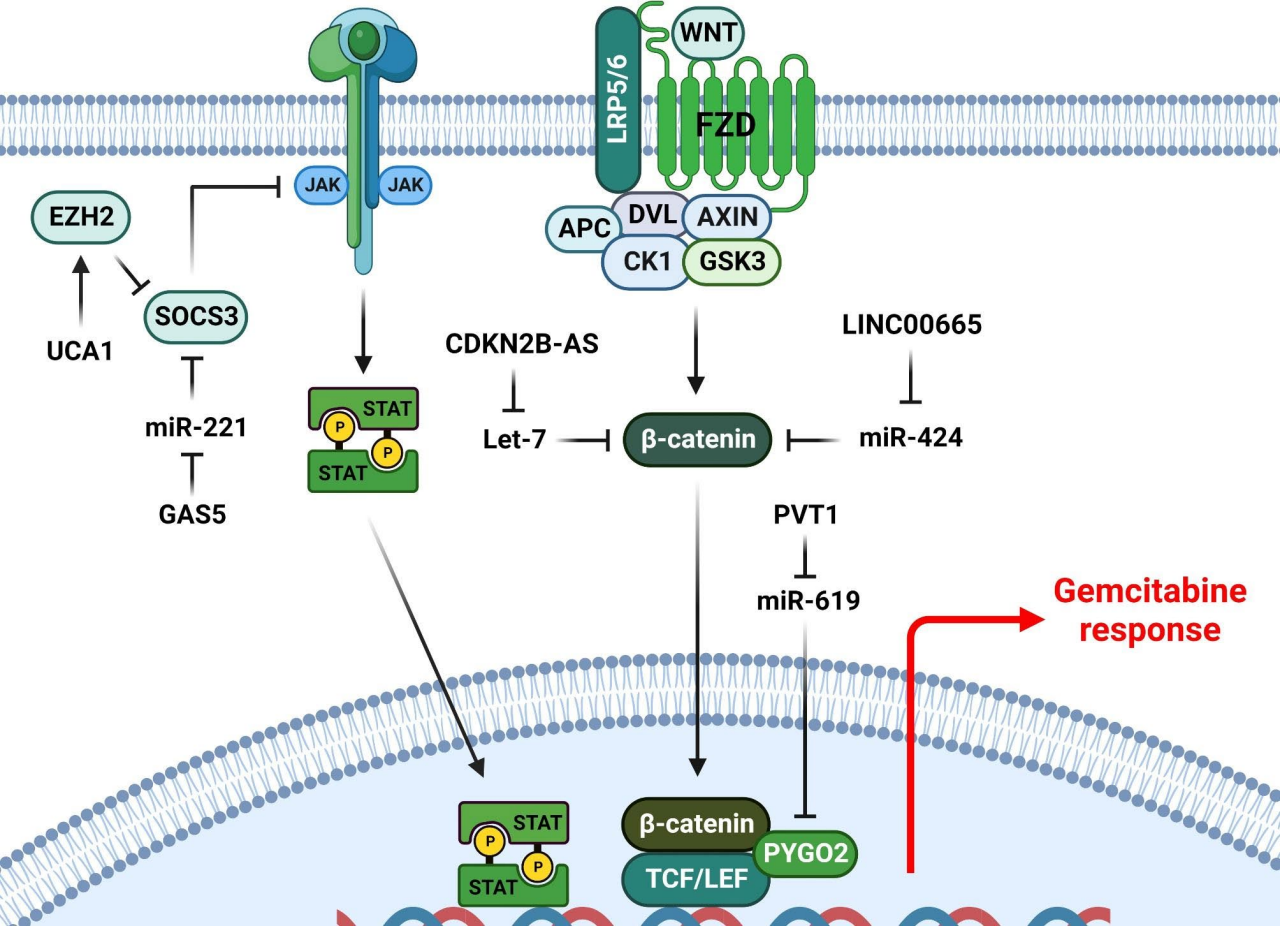
LncRNAs have an important function in GEM response of tumor cells by regulation of WNT and JAK/STAT signaling pathways. (Created with BioRender.com)

MAPK is a cascade of three kinases that can be activated by a variety of input signals such as cytokines, hormones, growth factors, and stress signals. It finally functions as a mitogen by ERK activation or stress response through JNK and p38. Therefore, MAPK/ERK has an oncogenic function by the induction of cell proliferation, migration, and drug resistance [50]. LncRNAs have a pivotal function in GEM response of tumor cells by regulation of MAPK/ERK signaling (Fig. 1). TUG1 up regulation has been shown in PDAC tissues in comparison with the normal margins. It promoted the GEM resistance in PDAC cells by ERK phosphorylation [51]. HDGF is a heparin-binding growth factor that promotes cell proliferation by the MAPK and PI3K pathways [52]. HCP5 up regulation was indicated in GEM-resistant PC tissues that were correlated with poor survival. HCP5 increased the GEM resistance by miR-214-3p/HDGF axis in PC cells [53].

## Wnt/β-catenin, TGF-b, and JAK/STAT signaling pathways

WNT pathway is essential for cell proliferation, tissue homeostasis, and apoptosis. Wnt ligands bind to FZD/LRP receptors that promote β-catenin to enter the nucleus to stimulate Wnt target genes expression via binding with TCF/LEF transcription factors and BCL9L and PYGO2 co-activators [54, 55]. LncRNAs have an important function in GEM response of tumor cells by regulation of WNT signaling (Fig. 2). Solid tumors recruit autophagy to overcome hypoxia, ischemia, radiotherapy, and chemotherapy [56, 57]. ATG14 has an important role in the autophagosome formation [58]. PVT1 has been shown to promote GEM resistance in pancreatic tumor cells by increasing WNT and autophagic activities. PVT1 plays a critical role in modulating the GEM resistance of PC cells via the miR-619-5p/Pygo2 and ATG14 axes [59]. LINC00665 up regulation has been found in GEM-resistant cholangiocarcinoma (CCA) cells that was correlated with the prognosis and chemotherapy resistance of CCA patients. LINC00665 sponged miR-424-5p to regulate BCL9L expression and WNT activation. Silencing of the LINC00665 reduced GEM-induced EMT and stemness in resistant CCA cells by decreasing β-Catenin nucleus translocation and BCL9L down regulation [60]. There was CDKN2B-AS up regulation in bladder urothelial carcinoma (BUC) tissues that was correlated with higher grades. CDKN2B-AS increased the GEM resistance of BUC via Let-7 sponging to activate the CTNNB1 [61].

TGF-β signaling is a key pathway in regulation of cell proliferation, apoptosis, and drug resistance. It is initiated by the TGF-β binding with TβR receptors which phosphorylates and activates TβR. Then activated TβR phosphorylates the R-Smad and Smad2/3 to form a complex with Smad4. Subsequently, Smad complexes enter to the nucleus to regulate the TGF-β target genes [62]. Smad4/R-Smad is activated by the TGF-β receptor to modulate the expression of genes involved in angiogenesis and chemo resistance [63, 64]. Smad4 knockdown induced cetuximab-resistance via the MAPK pathway targeting. Smad4 also induced the angiogenesis in ovarian cancer and increased the tumor development [65]. Smad4 acts as a tumor suppressor and inhibits the proliferation of tumor cells via Smad4/R-Smad complex. SNHG16 over expression has been found in GEM-resistant PC cells. SNHG16 recruited the EZH2 to catalyze H3K27me3 and suppress the Smad4, resulted in promotion of the AKT-mediated GEM-resistant PC cells [66]. Cancer stem-like cells (CSCs) are a sub-population of chemo-resistant tumor cells that are responsible for tumor recurrence [67, 68]. NF90 is an RNA-binding protein that has a significant role in the stabilization, turnover, and translation of various mRNAs [69–71]. TGFβ1 as an essential cytokine that induces EMT is highly correlated with the CSCs features [72]. It has been found that CSCs were highly abundant in UBC upon GEM treatment. TGFβ1 was up regulated in GEM-treated UBC cells to deregulate the LET/NF90/miR-145 signaling cascade, resulting in increased chemo resistance. LET down regulation was associated with NF90 protein stability, decreased biogenesis of miR-145, and increased stemness markers. The miR-145 targeted KLF4 and HMGA2 in UBC cells to inhibit stemness of tumor cells [73].

**Figure 3:**
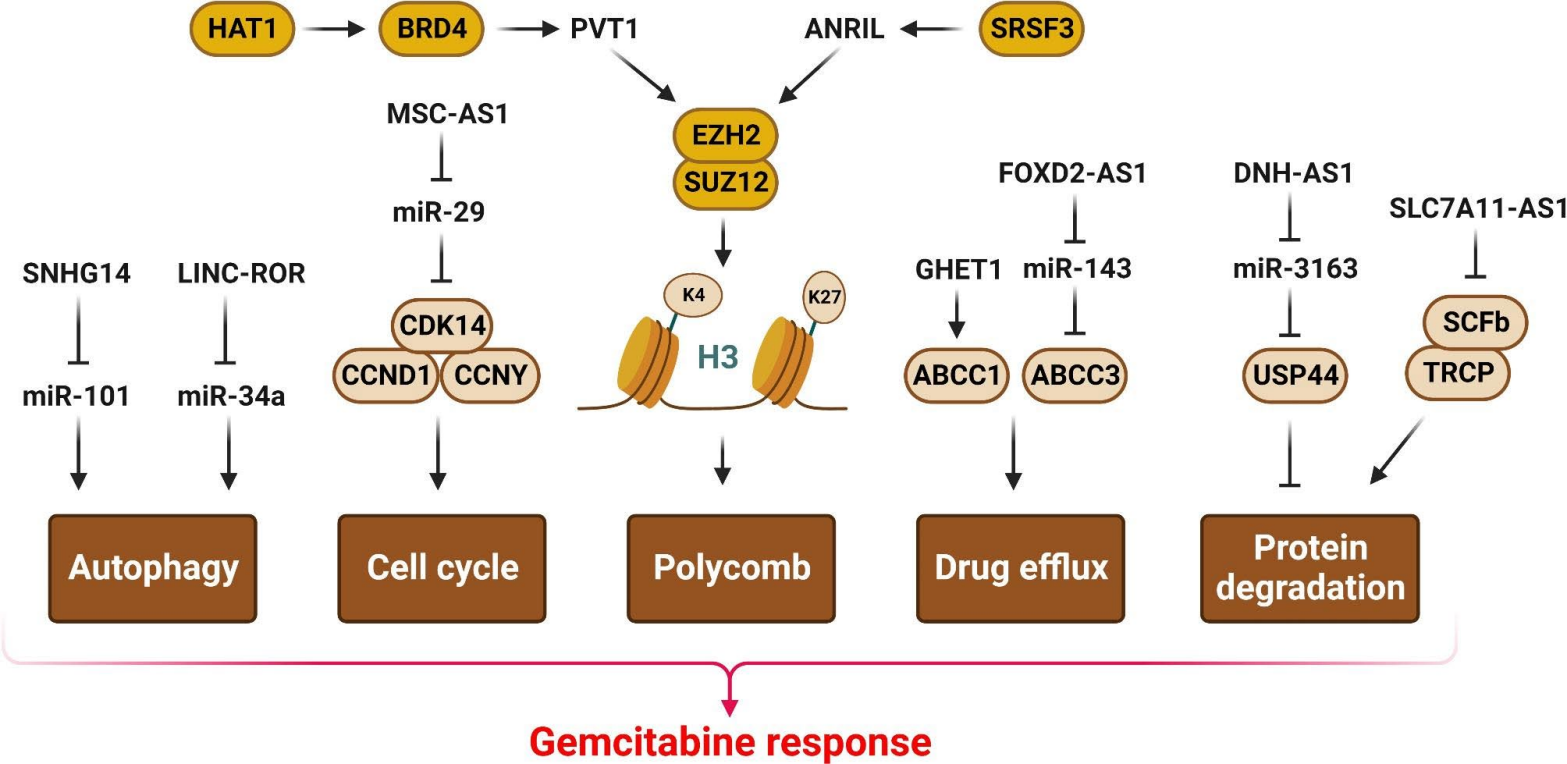
LncRNAs are involved in GEM response by regulation of structural proteins, autophagy, drug efflux, and cellular metabolism. (Created with BioRender.com)

The JAK/STAT signaling pathway is the main way to receive cytokines such as interleukins, interferons, and colony-stimulating factors. Therefore, it is involved in regulation of hematopoiesis, inflammation, and apoptosis. JAK/STAT signaling is initiated by cytokine binding to their receptors that recruits JAKs. Subsequently, activated JAKs promote the phosphorylation and dimerization of STATs that finally enter to the nucleus to regulate the JAK/STAT target genes. This pathway can be negatively regulated by the PIAS, SOCS, and PTPs. SOCS family inhibits the recruitment of STAT and JAK kinase activity [74]. LncRNAs have a pivotal role in GEM response of tumor cells by regulation of JAK/STAT signaling (Fig. 2). GAS5 suppressed GEM resistance in PC cells via miR-221/SOCS3 axis [75]. UCA1 suppressed SOCS3 via EZH2 recruitment in PC cells, thus promoting malignant traits and Gem resistance in pancreatic tumor cells [76].

## Transcription factors

Transcription factors are mainly the final effectors of various signaling pathways to regulate the expression of genes involved in cellular responses toward the intra and extra cellular stimuli. Therefore, they can be also involved in drug response of tumor cells. It has been shown that lnRNAs can affect the Gem response in tumor cells by regulation of transcription factors. C-MYC is an oncogenic transcription factor involved in cellular metabolism, proliferation, and drug resistance [77]. PVT1 plays a pivotal role in cancer through co-overexpression with the Myc [78, 79]. It has been identified that GEM triggers PVT1 processing into miRNAs through modulating the activity of the microprocessor in PC cells. After GEM exposure, the miR-1207 was upregulated in PC cells and inhibited oncogenic signaling by MYC targeting. PVT1 processing was mediated by Drosha/DGCR8 and that GEM modulated miRNA processing via Drosha and DGCR8 targeting [80]. FOXO3 belongs to the Forkhead box transcription factors that is negatively regulated by PI3K/AKT and MAPK/ERK pathways [81, 82]. It promotes apoptosis by up regulation of pro-apoptotic factors such as Bim and FasL [83]. FOXO3 inactivation by AKT confers 5-FU resistance [84]. There was LINC01714 down regulation in CCA that was related with poor prognosis. LINC01714 inhibited the migration and aggressiveness of CCA tumor cells, while promoted GEM sensitivity via FOXO3 inhibition [85]. The Bromodomain-containing Protein 4 (BRD4) is a transcriptional regulator by binding to the acetylated histones that is involved in modulation of cell proliferation and invasion [86, 87]. BRD4 is an important DNA repair regulator, and its inhibition has antitumor effects on various cancers. It also promotes the chemo sensitivity to GEM treatment [87, 88]. p21 is a negative regulator of cell-cycle progression, causing CCNB1 degradation to maintain cell-cycle arrest during the G2/M phase [89, 90]. Chk1 is also a ser/thr kinase that regulates cell-cycle progression during the G2/M phase [91]. LINC00346 improved the pancreatic tumor cell proliferation and colony formation. LINC00346 down regulation induced G2/M cell-cycle arrest in pancreatic tumor cells via p21 expression and Chk1 phosphorylation. It sponged miR-188-3p to enhance PC growth and GEM resistance via BRD4 targeting [92]. The HOX family belongs to the homeobox genes that encode transcriptional regulators to control cell proliferation and differentiation [93]. There were HOTTIP up regulations in human PC tissues and cell lines, which promoted tumor cell proliferation, EMT, and invasion. HOTTIP also increased GEM resistance via HOXA13 targeting [94]. High mobility group (HMG) is a family of non-histone DNA-binding proteins that are participated in regulation of transcription, DNA repair, and nucleosome assembly [95]. It was discovered that ANRIL down regulation inhibited PC cell growth and invasion and decreased GEM resistance via miR-181a targeting to regulate HMGB1-induced cell autophagy. MiR-181a activated autophagy via reducing LC3 I/II and increasing Beclin1 [96].

### Polycomb and RNA binding proteins

The majority of PC patients are detected at advanced stages, and there are limited surgical choices and a poor prognosis for these patients. Tumor recurrence and drug resistance are the most common causes of poor survival rate in PC patients [97]. GEM substitutes cytidine during DNA replication and blocks the production of deoxyribonucleotides to suppress pancreatic tumor cell growth [98]. The serine- and arginine-rich (SR) proteins belong to RNA-binding protein family, which regulates alternative splicing [99]. Serine/arginine-rich splicing factor 3 (SRSF3) is a SR protein family member that modulates cell senescence through detecting the alternative terminal exon [100, 101]. Messenger RNA metabolic processes from splicing to translation are regulated by m6A modification [102]. EZH2 as the catalytic member of Polycomb repressive complex 2 (PRC2) catalyzes the H3K27me3 to inhibit gene expression [103, 104]. LncRNAs are involved in GEM response by EZH2 promoter recruitment and transcriptional regulation of target genes (Fig. 3). There was SRSF3 up regulation in PC tissues that was correlated with GEM resistance and poor prognosis. SRSF3 modulated ANRIL exon inclusion, while improved exon skipping was observed by SRSF10 in PC cells. The m6A modification of ANRIL regulated its splicing in these cells. SRSF3 enhanced GEM resistance through ANRIL expression by constructing a complex with Ring1b and EZH2 that led to increased DNA homologous recombination repair [105]. HAT1 is a B-type histone acetyltransferase that regulates histone H4 N-terminus acetylation. Acetyl molecules can be present on cellular protein lysine residues as an epigenetic modulator [106]. Protein acetylation is a critical regulator of replication-dependent chromatin assembly, DNA damage repair, and gene expression [107, 108]. Mutated HAT1 enhances drug resistance in tumor cells. HAT1 improved liver tumor cell growth and caused cisplatin resistance [109]. EZH2 deregulation was correlated with GEM resistance via down regulation of p27Kip1 [110]. HAT1 silencing increased GEM sensitivity in PC cells via PVT1/EZH2 complex targeting. It also enhanced GEM resistance in PC cells by facilitating BRD4 binding to the PVT1 promoter and increasing PVT1 expression. Moreover, HAT1 protected EZH2 by inhibiting BRD4 from attaching to the N-terminal domain of EZH2 [111]. Cancer stem cells (CSCs) have a high self-renewal capacity that is associated with chemo resistance in PDAC tumors [112, 113]. It has been hypothesized that standard chemotherapy reduces the tumor mass by targeting the proliferating PDAC cells, while fails to target the CSCs that results in treatment failure [114]. Curcumin is a well-known suppressor of several oncogenes including ERK, AKT, and EZH2 in PDACs [115, 116]. PVT1 was up regulated in GEM-resistant PDAC cells. Curcumin also targeted CSCs and decreased the spheroid-forming capacity of GEM-resistant PDAC cells. Curcumin improved GEM sensitivity in pancreatic tumor cells via EZH2 blocking and its downstream target PVT1 [117]. GEM is a first-line chemotherapeutic agent that has been shown to enhance patient survival in un-resectable gallbladder cancer (GBC) patients [118, 119]. Nevertheless, only 36% of GBC patients benefit from GEM treatment that is related to the drug resistance [120]. NONO is a critical RNA binding protein in different types of cancers [121, 122]. NONO increased oxaliplatin sensitivity in CRC cells indicating that NONO has pivotal role in drug resistance of tumor cells [123]. There was SSTR5-AS1 up regulation in GBC tissues that was associated with poor OS in GBC patients. SSTR5-AS1 directly regulated the NONO protein, which resulted in increased GEM resistance in these patients [124].

## Autophagy and cell cycle regulation

Autophagy is a critical biochemical process that preserves cellular homeostasis and survival by removing and recycling unneeded and damaged cellular materials and organelles. As a multidimensional catabolic process, autophagy has been preserved throughout the evolution. Drug resistance reduces the apoptotic response of tumor cells via abnormal cell autophagy [125–127]. Autophagy is a critical regulator of tumor development and cancer therapy via promoting cell survival [56]. PI3K pathway is one of the main regulators of autophagy in response to ROS levels. PI3Kα subunit is a suppressor of autophagy by AKT/mTORC2 axis under moderate ROS levels [128]. Regarding the role of autophagy in drug response, lncRNAs can be involved in autophagy mediated GEM response in tumor cells (Fig. 3). Inhibition of autophagy sensitizes the cancer cells to GEM, while decreasing the stemness of pancreatic cancer cells [129]. It has been shown that the SNHG14 enhanced PDAC cells progression and autophagy mediated GEM resistance via miR-101 sponging [130]. Neoadjuvant chemotherapy including capecitabine, bevacizumab, GEM, and taxanes, has improved the prognosis for patients with metastatic breast cancer [131]. GEM regulates autophagy via Beclin1, ATG16L1, and LC3, and promotes apoptosis in tumor cells via Bcl-2 and Bax [132–135]. Apoptosis is a key mechanism that controls tissue homeostasis, whereas autophagy is an essential biological process that eliminates damaged cellular components [136]. Linc-ROR suppressed histone H3 acetylation in the miR-34a promoter to reduce GEM-mediated apoptosis and autophagy, resulting in miR-34a down regulation. LincROR inhibition induced the LC3-II, Beclin1, and NOTCH1 expressions, while reduced p62 expression in breast tumor cells [137]. HOST2 down regulation reduced GEM resistance to promote apoptosis in pancreatic tumor cells [138]. Cyclin-dependent kinases (CDKs) have key roles in regulation of the cell cycle progression [139]. CDK14 regulates the cell cycle progression by interacting with CCND3 and CCNY. It also stimulates the WNT pathway by targeting its downstream proteins [140]. There were MSC-AS1 up regulations in PAAD and PDAC tissues that was directly correlated with CDK14 up regulation and poor prognosis. There was significant down regulation of miR-29b-3p in the PDAC tissues that was correlated with a poor outcome in PDAC patients. MSC-AS1/miR-29b-3p axis regulated CDK14-mediated cell proliferation and GEM-mediated apoptosis in PDAC cells [141].

## ABC transporters and structural proteins

Structural proteins have not a direct role in transcriptional regulation. However, they have critical roles in regulation of cellular metabolism, membrane traffic, and cell migration. LncRNAs are also involved in GEM response by regulation of structural proteins, ABC transporters, and cellular metabolism (Fig. 3). Multidrug resistance-associated protein 1 (MRP1) belongs to the ABC transporter protein family that is primarily involved in the transport of multiple intracellular and extracellular complexes. As an efflux pump, it removes the chemotherapy drugs from tumor cells. MRP1 promotes the chemotherapeutic resistance in tumor cells via lowering intracellular drug concentration. It has been reported that there was GHET1 up regulation in BC that was associated with higher grades and muscle invasion. GHET1 enhanced the GEM resistance in BC via ABCC1 up regulation [142]. There was a dose-dependent association between FOXD2-AS1 up regulation and GEM-resistance of bladder tumor cells. FOXD2-AS1 enhanced the GEM-resistance of bladder cancer via miR-143/ABCC3 targeting [143].

A low intracellular reactive oxygen species (ROS) level is required to maintain the self-renewal of tumor cells [144]. GEM triggers the cell apoptosis via ROS production [145]. Chemo resistant tumor cells have a strong antioxidant system to regulate the excessive generation of ROS in order to survive under oxidative stress [146]. NRF2 is the major mediator of redox hemostasis that is typically up regulated in CSCs [147]. SKP1-Cul1-Rbx1 (SCFb-TRCP) E3 complex participates in the proteasomal degradation of NRF2 [148, 149]. SLC7A11-AS1 up regulation and its association with drug resistance was demonstrated in GEM-resistant PDAC cells. SLC7A11-AS1 preserved NRF2 via inhibiting ubiquitination mediated by SCFb-TRCP, which is essential to sustain the self-renewal and chemo resistance of PDAC cells. SLC7A11-AS1 up regulation was contributed with poor prognosis in PDAC patients [150]. USP44 as a deubiquitinase has pivotal roles in regulation of spindle assembly and anaphase onset by deubiquitination of CDC20 that is an inhibitor of APC/C. There was DNH-AS1 down regulation in GEM resistant PC cells which was associated with prognosis. The m6A methylase knockdown has been demonstrated to reduce the levels of DBH-AS expressions in PC cells. DNH-AS1 acted as a sponge for miR-3163 to up regulate the USP44 that was involved in the GEM absorption in tumor cells [151].

Twinfilin 1 (TWF1) as an inhibitor of the actin polymerization is involved in regulation of cell migration, drug sensitivity, and tumor progression [152, 153]. Inhibition of the SBF2-AS1 decreased the levels of TWF1 expressions via miR-142-3p sponging to increase GEM resistance in pancreatic tumor cells [154]. EMT is one of the pathophysiological cellular processes that is involved in regulation of embryogenesis, tumor metastasis, and drug resistance [155]. It is orchestrated by the up regulation of mesenchymal proteins such as CDH2 and VIM, while down regulation of CDH1 epithelial marker that finally changes the epithelial to mesenchymal phenotype to facilitate the tumor cell migration [156]. MVIH down regulation increased the expression of CDH1 while decreased the Vimentin expression, which induced GEM sensitivity in gastric tumor cells [157].

## Conclusions

LncRNAs as the regulators of cellular mechanisms such as cell proliferation and apoptosis have a critical role in chemotherapeutic response. In the present review, we discussed the role of lncRNAs in GEM response. It has been shown that lncRNAs mainly induced GEM resistance through the regulation of transcription factors, autophagy, polycomb complex, and signaling pathways. Therefore, lncRNAs can be introduced as the non-invasive prognostic markers to predict the GEM response and improve the therapeutic strategy among cancer patients. Despite the clinical advantages of lncRNA as non-invasive diagnostic and prognostic markers in cancer patients, some limitations have slowed down the entry of these markers into the clinic. One of the upcoming limitations is the requirement to examine the levels of lncRNA expressions in the serum of cancer patients. Animal studies are also needed to introduce lncRNA as the prognostic markers and therapeutic targets. However, the majority of reports on the relationship between lncRNAs and GEM response are limited to the in-vitro studies and assessment of their expression levels in tumor tissues. Another existing limitation is the complexity of cellular processes affected by lncRNAs, which can be related to their ability to bind with DNA, RNA, and proteins that can affect a wide range of cellular processes. Therefore, detailed studies on the molecular mechanisms of lncRNAs can play a very important role in preclinical studies to pave the way to introduce lncRNAs as the reliable diagnostic and prognostic markers in the clinic.

## Data Availability

The datasets used and/or analyzed during the current study are available from the corresponding author on reasonable request.

## Abbreviations

BUC: Bladder urothelial carcinoma
BRD4: Bromodomain-containing Protein 4
CSCs: Cancer stem cells
CCA: Cholangiocarcinoma
ceRNAs: Competing endogenous RNAs
CDKs: Cyclin-dependent kinases
EMT: Epithelial?mesenchymal transition
GBC: Gallbladder cancer
GEM: Gemcitabine
lncRNAs: Long non-coding RNAs
MRP1: Multidrug resistance-associated protein 1
NSCLC: Non?small cell lung carcinoma
PC: Pancreatic cancer
PRC2: Polycomb repressive complex 2
ROS: Reactive oxygen species
RTKs: Receptor tyrosine kinases
SR: Serine- and arginine-rich
SRSF3: Serine/arginine-rich splicing factor 3
TWF1: Twinfilin 1
